# The Roman Bridge: a "double pulley – suture bridges" technique for rotator cuff repair

**DOI:** 10.1186/1471-2474-8-123

**Published:** 2007-12-18

**Authors:** Francesco Franceschi, Giuseppe Umile Longo, Laura Ruzzini, Giacomo Rizzello, Nicola Maffulli, Vincenzo Denaro

**Affiliations:** 1 Department of Orthopaedic and Trauma Surgery, Campus Biomedico University, Via Alvaro del Portillo, 200, 00128 Trigoria, Rome, Italy; 2 Department of Trauma and Orthopaedic Surgery, University Hospital of North Staffordshire, Keele University School of Medicine, Stoke on Trent, ST4 7LN UK

## Abstract

**Background:**

With advances in arthroscopic surgery, many techniques have been developed to increase the tendon-bone contact area, reconstituting a more anatomic configuration of the rotator cuff footprint and providing a better environment for tendon healing.

**Methods:**

We present an arthroscopic rotator cuff repair technique which uses suture bridges to optimize rotator cuff tendon-footprint contact area and mean pressure.

**Results:**

Two medial row 5.5-mm Bio-Corkscrew suture anchors (Arthrex, Naples, FL), which are double-loaded with No. 2 FiberWire sutures (Arthrex, Naples, FL), are placed in the medial aspect of the footprint. Two suture limbs from a single suture are both passed through a single point in the rotator cuff. This is performed for both anchors. The medial row sutures are tied using the double pulley technique. A suture limb is retrieved from each of the medial anchors through the lateral portal, and manually tied as a six-throw surgeon's knot over a metal rod. The two free suture limbs are pulled to transport the knot over the top of the tendon bridge. Then the two free suture limbs that were used to pull the knot down are tied. The end of the sutures are cut. The same double pulley technique is repeated for the other two suture limbs from the two medial anchors, but the two free suture limbs are used to produce suture bridges over the tendon, by means of a Pushlock (Arthrex, Naples, FL), placed 1 cm distal to the lateral edge of the footprint.

**Conclusion:**

This technique maximizes the advantages of two techniques. On the one hand, the double pulley technique provides an extremely secure fixation in the medial aspect of the footprint. On the other hand, the suture bridges allow to improve pressurized contact area and mean footprint pressure. In this way, the bony footprint in not compromised by the distal-lateral fixation, and it is thus possible to share the load between fixation points. This maximizes the strength of the repair and provides a barrier preventing penetration of synovial fluid into the healing area of tendon and bone.

## Background

Rotator cuff surgery aims to provide tendon fixation secure enough to hold the repaired tendon in place until biological healing occurs [1]. Healing of repaired rotator cuff tendons will be helped by appropriate restoration of the anatomic footprint and constructs providing adequate compression of the tendon on the footprint itself [2,3].

With advances in arthroscopic surgery, many techniques have been developed to increase the tendon-bone contact area, reconstituting a more anatomic configuration of the rotator cuff footprint and providing a better environment for tendon healing [3-10].

We present an arthroscopic rotator cuff repair technique which uses suture bridges to optimize rotator cuff tendon-footprint contact area and mean pressure.

The Roman Bridge (double pulley – suture bridges) technique maximizes the advantages of the two techniques. The double pulley technique provides an extremely secure fixation in the medial aspect of the footprint [8,11,12]. The suture bridges allow to improve pressurized contact area and mean footprint pressure, to not compromise the bony footprint by the distal-lateral fixation, produce a low-profile repair, share the load between fixation points, which maximizes the strength of the repair and to provide a barrier of synovial fluid from the healing zone involving tendon and bone [5-7].

## Results

### Arthroscopic technique

All procedures described in the present article were approved by the Local Ethics Committee of the Campus Biomedico University, Rome, Italy, and all patients gave their written consent. Patients undergo brachial plexus block and are placed in a lateral decubitus position. The arm is suspended at approximately 45° of abduction and 20° of forward flexion. Distraction of the shoulder joint is accomplished with 4.5 to 6.5 kg of traction. Four to six portals are used. A posterior portal is produced, and the arthroscope is inserted into the glenohumeral joint. A diagnostic arthroscopy is performed to evaluate the extent of the rotator cuff tear, lesions of the biceps tendon, and other associated lesions. The main subacromial portals are the postero-lateral viewing, the antero-lateral, and the lateral working portal, with an 8.25 mm cannula. To control bleeding, we use radiofrequency, adrenalin admixture to the irrigation fluid, and ask the anesthesiologist to lower the systolic blood pressure to 90 mm Hg if possible. An arthroscopic pump maintains fluid pressure at 40 mmHg, increasing it temporarily on demand.

A spinal needle is introduced percutaneously to determine the precise location for placement of the antero-lateral portal produced approximately 2 to 3 cm anterior and lateral to the antero-lateral corner of the acromion. If the subscapularis tendon is involved, an anterior mid-lateral portal is produced just superior to the lateral half of the subscapularis tendon. The lateral portal is used to mobilize the rotator cuff back to its bony insertion. The mobility of the rotator cuff is assessed.

Using a burr through the lateral portal, the footprint of the greater tuberosity is abraded.

**Figure 1:**
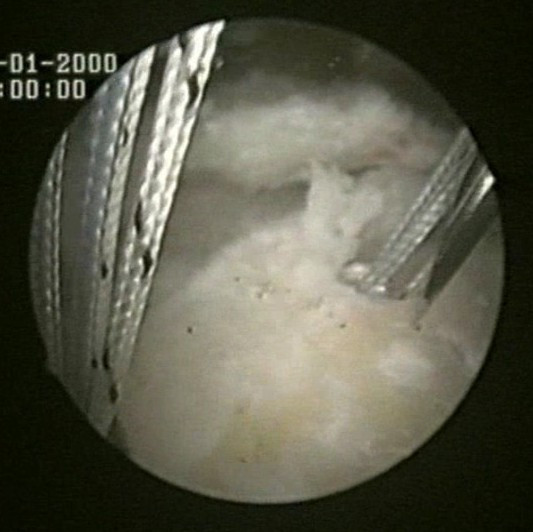
Two medial row suture Bio-Corkscrew anchors (Arthrex, Naples, FL), which are double-loaded with No. 2 FiberWire sutures (Arthrex, Naples, FL), are placed in the medial aspect of the footprint, just lateral to the articular surface of the humeral.

Two medial row 5.5-mm Bio-Corkscrew suture anchors (Arthrex, Naples, FL), which are double-loaded with No. 2 FiberWire sutures (Arthrex, Naples, FL), are placed in the medial aspect of the footprint, just lateral to the articular surface of the humeral head (Fig 1). The first anchor is placed in the anteromedial aspect of the footprint. The second anchor is placed approximately 1.5 to 2 cm posterior to the first anchor.

**Figure 2:**
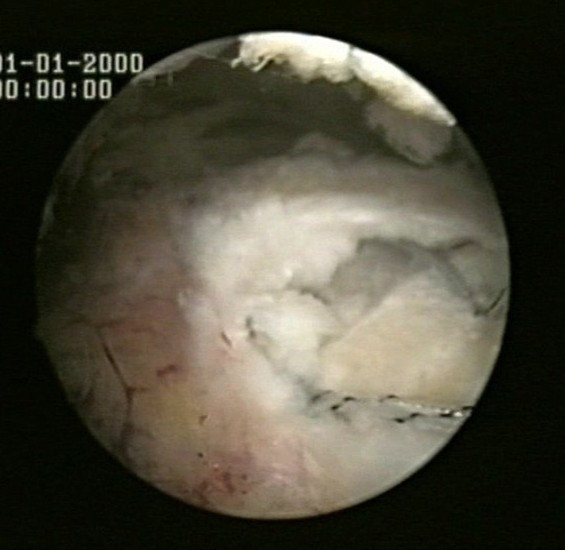
Two suture limbs from each anchor are sequentially passed through two single points in the rotator cuff.

**Figure 3:**
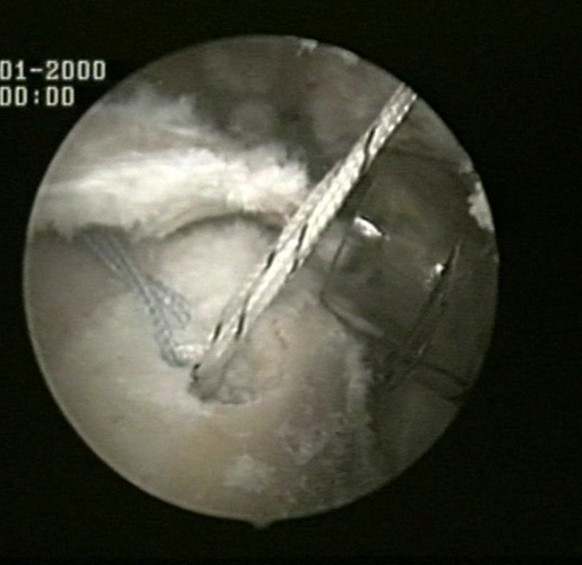
Two suture limbs from each anchor are sequentially passed through two single points in the rotator cuff.

Two suture limbs from a single suture are both passed through a single point in the rotator cuff by means of a Penetrator or a BirdBeak suture passer (Arthrex), producing two points of fixation in the tendon, with a tendon bridge between them (Fig 2, 3). This is performed for both anchors.

**Figure 4:**
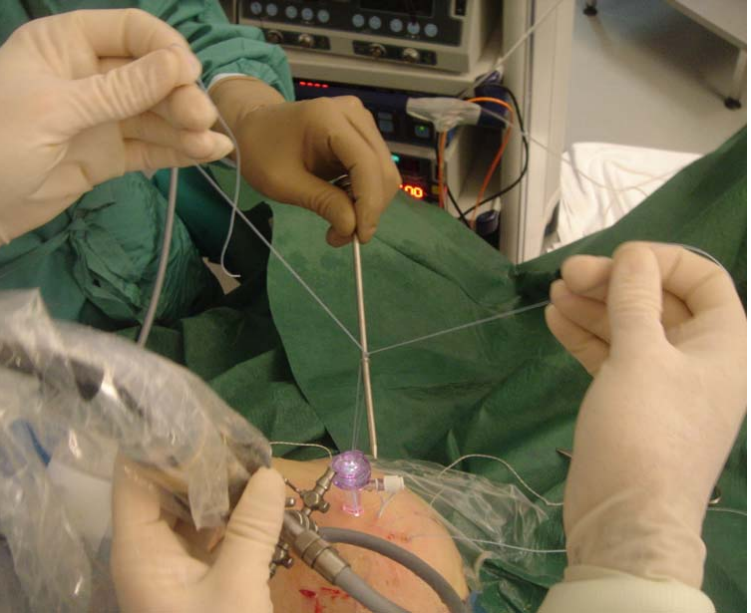
A suture limb is retrieved from each of the medial anchors through the lateral portal, and manually tied as a six-throw surgeon's knot over a metal rod.

**Figure 5:**
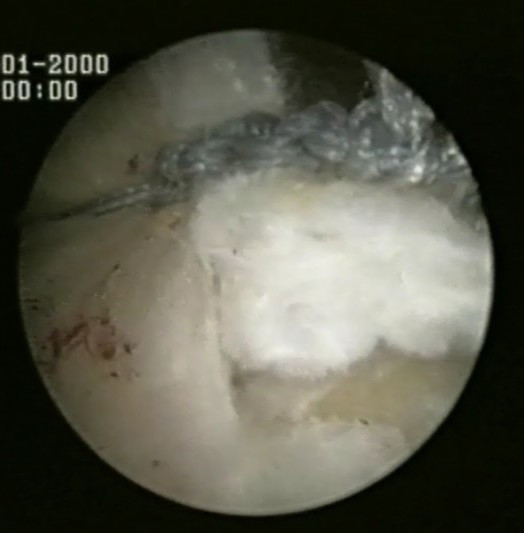
The two free suture limbs are pulled to transport the knot over the top of the tendon bridge.

**Figure 6:**
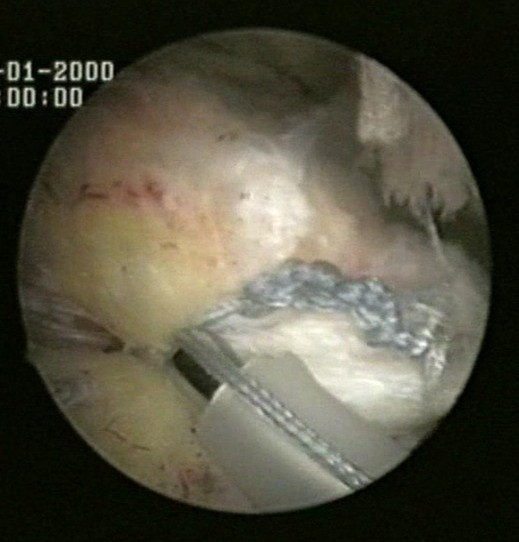
The two free suture limbs are tied with a Surgeon's Sixth Finger (Arthrex, Naples, FL) knot pusher as a static, nonsliding knot.

The medial row sutures are tied using the double pulley technique. A suture limb is retrieved from each of the medial anchors through the lateral portal, and manually tied as a six-throw surgeon's knot over a metal rod (Fig 4). A tendon grasper introduced through a lateral portal is used to grasp the medial aspect of the rotator cuff tendon, which is pulled laterally toward the bone bed. The two free suture limbs are pulled to transport the knot over the top of the tendon bridge (Fig 5). This technique is called the "double-pulley" technique, because the eyelets of two suture anchors are used like pulleys to bring the knots down onto the cuff. Then the two free suture limbs that were used to pull the knot down are tied with a Surgeon's Sixth Finger (Arthrex, Naples, FL) knot pusher as a static, nonsliding knot (Fig 6). This produces a double mattress suture between the two medial anchors. The end of the sutures are cut.

The same double pulley technique is repeated for the other two suture limbs from the two medial anchors.

The other two suture limbs of the same colour from each anchor are sequentially passed through two single points in the rotator cuff, producing two points of fixation in the tendon, with a tendon bridge between them.

**Figure 7:**
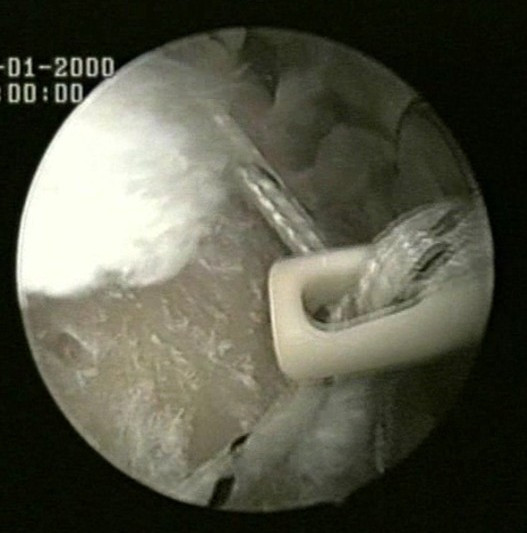
The two free suture limbs are used to produce suture bridges over the tendon, by means of a Pushlock (Arthrex, Naples, FL).

**Figure 8:**
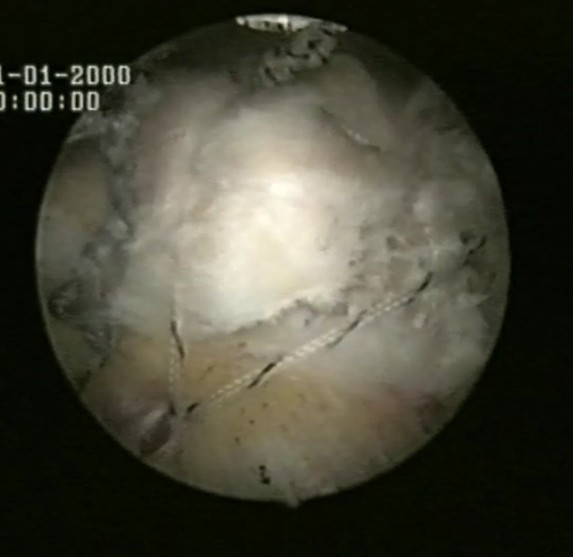
The final results.

The suture limb is retrieved from each of the medial anchors through the lateral portal, and manually tied as a six-throw surgeon's knot over a metal rod. The two free suture limbs are pulled to transport the knot over the top of the tendon bridge. Then the two free suture limbs are used to produce suture bridges over the tendon, by means of a Pushlock (Arthrex, Naples, FL), placed 1 cm distal to the lateral edge of the footprint centered relative to the medially placed suture anchors anterior to posterior (Fig 7, 8).

**Figure 9:**
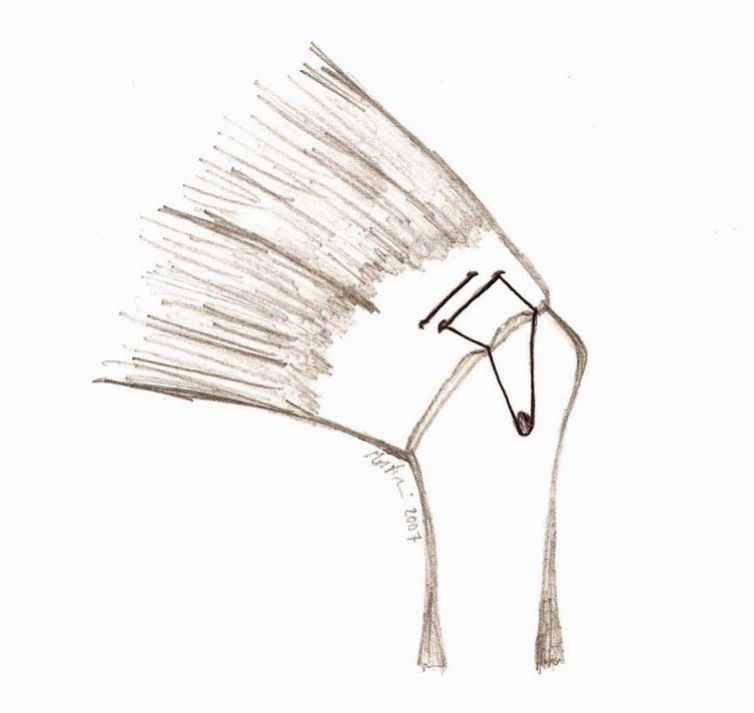
A schematic drawing of the Roman Bridge technique.

The final result is what we call the Roman Bridge: the combination of double pulley and suture bridges techniques (Fig 9).

### Post-operative management

The arm is supported in a sling with an abduction pillow for 6 weeks. Active elbow flexion and extension are allowed, but terminal extension is restricted. Passive external rotation is started from the first day after surgery, and maintained within a comfortable range. Overhead stretching is restricted until 6 weeks post-operatively to avoid damaging the repair. At six weeks, the sling is removed, and overhead stretching with a rope and pulley are started. Isoinertial strengthening and rehabilitation of the rotator cuff, deltoid and scapular stabilizers are initiated at 10 or 12 weeks after the operation. Rehabilitation is continued for 6 months. Heavy manual work and overhead activities are allowed after a good restoration of shoulder strength, which normally occurs 6 to 10 months after surgery.

## Discussion

To improve outcomes after rotator cuff repair, the biology of repaired rotator cuff insertion needs to be respected [13]. Recently, attempts have been made to enhance the tendon-to-bone healing process from a biological standpoint [13,14]. As we re-attach tendinous tissue to bone, theoretically only the re-constitution of enthesial fibrocartilage [15] would guarantee an optimal outcome [13,16]. To improve rotator cuff tendon healing, rotator cuff fixation strength has been extensively studied [14,17]. To optimize the healing process, it seems to be important to attempt restoration of the original anatomy of the insertion of the rotator cuff, which would provide larger area for bony incorporation and healing, and to develop constructs that provide increased compression of the tendon on the footprint which may affect the mechanical strength and function of the repaired tendon [1-4,8,18-20]. This is especially important at the early stages of rehabilitation, when the tendon-bone interface is still weak, and complete functional recovery has yet to take place [19,21,22]. With the development of new biological enhancement techniques, it might prove important to maintain a large area of contact between tendon and bone, allowing more fibers to participate in the healing process.

Several arthroscopic techniques of rotator cuff repair have been proposed to develop constructs which provide increased compression of the tendon on the footprint, but controversy continues to exist for suture anchor repairs compared to the transosseous tunnel technique [1,3-8,18].

Many studies have shown comparable or superior initial fixation strength for suture anchor repairs. Apreleva et al [2] and Park et al [4] showed that an improved repair site area is obtained with a transosseous repair technique when compared to suture anchor techniques.

The suture tension for the transosseous technique provides a more direct tendon-to-bone compression vector. In contrast, the sutures for the suture anchor technique predominantly provide circumferential tension around the tendon but relatively little compression between tendon and bone [6,7]. Suture bridges provide significantly more compression compared with suture anchor techniques. Improved pressure characteristics with a transosseous technique may allow for improved tendon-to-bone healing and a lower persistent tear rate.

The transosseous-equivalent rotator cuff repair technique has been developed to optimize healing biology at a repaired rotator cuff tendon insertion [5-7]. This technique for arthroscopic repair of rotator cuff tears improves the contact area and the mean footprint pressures, without compromising the bony footprint by the distal-lateral fixation. It also produces a low-profile repair, sharing the load with the suture-bridge technique between fixation points, which maximizes the strength of the repair [2,5-7]. The repair involves inserting a medial row with suture anchors that utilize mattress repairs.

The double pulley technique provides extreme secure fixation in mobile crescent-shaped rotator cuff tears [8,11,12]. This technique is a "double-pulley" technique, because the eyelets of two sutures anchors are used like pulley to bring the knots down onto the rotator cuff. At the end of the procedure, a bridge of cuff tissue is compressed against the bone bed, producing a stable construct in the medial aspect of the footprint [8,11,12].

Our technique of "double pulley-suture bridges" repair maximizes the good point of two techniques. In addition to the strong medial fixation obtained by means of a double pulley, the sutures bridges improve compression contact area and mean footprint pressure, provide a barrier to the synovial fluid from the joint to the healing area of tendon and bone, and share the load between fixation points. By providing suture bridges of fixation, the number of points of fixation is increased, increasing the strength of the initial repair construct, and decreasing the load which each suture loop and knot must resist and the stress at each suture-cuff contact point [5-8].

The technique described in this manuscript may provide greater potential for osseous incorporation and healing at the tendon-bone interface by increasing the repair site area and thus greater ultimate strength of the repair compared with a single row suture anchor repair [2,13,21].

The ideal patient for a Roman bridge repair is one with medium U-shaped tear of the rotator cuff. This technique is not indicated for patients with retracted large and massive rotator cuff tear.

Our technique saves time when compared to a traditional procedure, as in the medial row the suture limbs from a single suture for each anchor are both passed through a single point in the rotator cuff. Also, the Pushlock in the lateral row does not require tying the knot.

Disadvantages of our technique include that, by the end of the procedure, one of the medial anchors may pull out, and this can not be identified by subacromial visualization. We therefore recommend glenohumeral inspection of the repair at the end of the procedure, although in our experience this has never been the case. Furthermore, the 6-throw surgeon's knot might cause irritation in the subacromial space. However, we never encountered this potential complication in our patients. Moreover, as any procedure aimed to restore the anatomical footprint of the rotator cuff, our technique is more expensive than a single row suture anchor repair, as it requires more suture anchors.

## Conclusion

Additional biomechanical and clinical investigations are needed. Nevertheless, the Roman Bridge "Double Pulley – Suture Bridges" repair is a viable option for the arthroscopic management of rotator cuff tears.

## Methods

Using a burr through the lateral portal, the footprint of the greater tuberosity is abraded.

Two medial row 5.5-mm Bio-Corkscrew suture anchors (Arthrex, Naples, FL), which are double-loaded with No. 2 FiberWire sutures (Arthrex, Naples, FL), are placed in the medial aspect of the footprint, just lateral to the articular surface of the humeral head (Fig 1).

Two suture limbs from a single suture are both passed through a single point in the rotator cuff by means of a Penetrator or a BirdBeak suture passer (Arthrex), producing two points of fixation in the tendon, with a tendon bridge between them (Fig 2, 3). This is performed for both anchors.

The medial row sutures are tied using the double pulley technique (Fig 4). A tendon grasper introduced through a lateral portal is used to grasp the medial aspect of the rotator cuff tendon, which is pulled laterally toward the bone bed.

The two free suture limbs are pulled to transport the knot over the top of the tendon bridge (Fig 5). Then the two free suture limbs that were used to pull the knot down are tied with a Surgeon's Sixth Finger (Arthrex, Naples, FL) knot pusher as a static, nonsliding knot (Fig 6). The same double pulley technique is repeated for the other two suture limbs from the two medial anchors.

The other two suture limbs of the same colour from each anchor are sequentially passed through two single points in the rotator cuff, producing two points of fixation in the tendon, with a tendon bridge between them.

The suture limb is retrieved from each of the medial anchors through the lateral portal, and manually tied as a six-throw surgeon's knot over a metal rod. The two free suture limbs are pulled to transport the knot over the top of the tendon bridge. Then the two free suture limbs are used to produce suture bridges over the tendon, by means of a Pushlock (Arthrex, Naples, FL), placed 1 cm distal to the lateral edge of the footprint centered relative to the medially placed suture anchors anterior to posterior (Fig 7, 8).

The final result is what we call the Roman Bridge: the combination of double pulley and suture bridges techniques (Fig 9).

## Competing interests

The author(s) declare that they have no competing interests.

## Authors' contributions

FF, UGL, NM and VD conceived the study. UGL, LR, and GR performed the review of the literature and wrote the initial draft. They also consented the patients whose photos are shown in this manuscript. FF, NM and VD advised on the practicalities of the surgery. All authors read and approved the final manuscript. No funding has been received for the study.

## Pre-publication history

The pre-publication history for this paper can be accessed here:


